# The role of philanthropy in shaping the market for near-vision spectacles

**Published:** 2026-03-12

**Authors:** Abigail Steinberg, Peter Holland

**Affiliations:** 1 Executive Director: Livelihood Impact Fund, Denver, USA.; 2 Chief Executive: International Agency for the Prevention of Blindness (IAPB), London, UK.

**Presbyopia is a global challenge that can be solved in our lifetime.**

**Figure:**
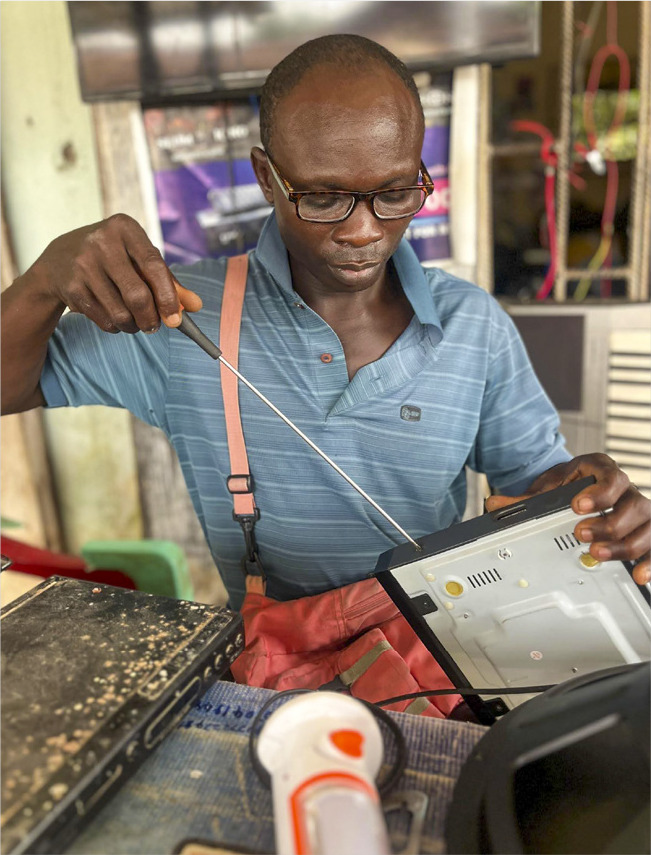
Near-vision spectacles restore vision and livelihoods. NIGERIA

© CLINTON HEALTH ACCESS INIATIVE CC BY-NC-SA 4.0

Few global health challenges are both sizable and solvable within a decade. Presbyopia, or age-related near-vision loss, is one such challenge. Over 1.8 billion people live with presbyopia, but an estimated 800 million lack access to the solution: low-cost near-vision spectacles (reading glasses) that are safe, effective, and can be self-selected - allowing people with presbyopia to farm, craft, read, write, tailor, and provide health care, among other tasks.

We believe providing affordable access to near-vision spectacles to 800 million people in the next 10 years is not only possible, but also solves a productivity and livelihoods issue: it could yield USD 1.05 trillion in productivity gains by 2050.^1^

The experience of restored near vision is transformative. However, many people are not aware of presbyopia, and do not realise that their vision can easily be improved by near-vision spectacles. As a result, normal market forces do not (yet) apply.

Philanthropy can play a critical role in helping to create that initial demand: by supporting subsidised or free access for people receiving their first pair of near-vision spectacles. Such initial ‘angel’ investment in presbyopia is highly effective and impactful and - along with policy changes and other efforts - contributes to longer-term market shaping and sustainability.

Research across India,^2^ Sierra Leone,^3^ Pakistan, and other countries shows that, once people begin using near-vision spectacles, uptake becomes ‘sticky’: usage rates remain well above 70% even after five years; in India and Pakistan, these rates are greater than 90%.^2^ Repurchasing rates (buying a second pair) reach 50-60%, even among those living at or just above the poverty line, at price points above USD 6 per pair. This demonstrates that - while subsidies or free distribution are essential to overcome the barrier of first-time use - many individuals are willing and able to purchase subsequent pairs once they see the value in near-vision spectacles: usually only once they experience, for themselves, the transformative effect this has on their sight and their lives. In this sense, philanthropy helps to ‘de-risk’ the market: it covers the cost of initial adoption and paves the way for sustainable, consumer-driven demand.

However, philanthropy alone cannot meet the scale of global need. Long-term sustainability depends on market-shaping strategies that ensure affordable, widely available spectacles. These strategies include strengthening supply chains to reduce the cost of manufacturing and distribution, supporting local entrepreneurs to deliver near-vision spectacles through community networks, pharmacies, and private outlets, and developing financing models such as community-driven savings tools, cross-subsidisation, or insurance coverage that make spectacles affordable for those with limited incomes. Policy and regulatory support are also essential, enabling ready-made near-vision spectacles to be sold via non-medical channels - thereby dramatically increasing the number of access points.

Encouragingly, we already see models that work, as described in other articles in this issue.

Experience from countries where near-vision spectacles are normalised, such as the United States and the United Kingdom, shows that, once people see near-vision spectacles as a routine part of ageing, awareness campaigns become almost unnecessary. People naturally seek out spectacles when they notice their near vision declining, having seen parents and grandparents do the same. This is the tipping point we aim to reach globally: once enough people have access, the behaviour becomes self-sustaining across generations (see pp. 15-17). Each pair of spectacles not only restores sight, but also builds the expectation that vision correction is normal and attainable.

Philanthropy is often drawn to ‘big, solvable problems,’ Guinea worm eradication being a famous example. Presbyopia and refractive error correction belong in this category. If we act now, we can bring near-vision correction to billions within a decade. If we delay, it could take 80-100 years to achieve universal access to spectacles at the current pace, with immeasurable social and economic costs. The opportunity is extraordinary: spectacles are among the most cost-effective health interventions in existence, with immediate impact on livelihoods, productivity, and quality of life. Studies show improved income and productivity among tea pickers,^4^ garment workers,^5^ and others^6^ once they have received near-vision spectacles. For philanthropy and health systems alike, this is a ‘best buy’ intervention.

Philanthropy is indispensable for getting spectacles onto faces for the first time. But the true promise lies in creating a sustainable, affordable market: one where access no longer depends on external grant dollars, but on locally rooted supply and demand. With the right mix of investment, policy, and entrepreneurship, presbyopia correction can move from being an overlooked global health issue to a solved one - within our lifetimes.
